# Clinical Audit to Assess Orthogeriatrician Input to the Management of Elderly Trauma Patients

**DOI:** 10.7759/cureus.65173

**Published:** 2024-07-23

**Authors:** Marwan Tahoun, Tom Collins, Rana Tahoun, Abdul Hadi Kafagi, Anand Pillai

**Affiliations:** 1 Trauma and Orthopaedics, Wythenshawe Hospital, Manchester University NHS Foundation Trust, Manchester, GBR; 2 Trauma and Orthopaedics, Countess of Chester Hospital, Chester, GBR

**Keywords:** clinical audit, boast, british orthopaedic association, orthogeriatrician, morbidity and mortality, fragility fractures, geriatric hip fracture, elderly trauma, orthogeriatrics

## Abstract

Objective: The primary objective of this study is to assess the adherence of our department to the British Orthopaedic Association's Standards for Trauma and Orthopaedics (BOAST) guidelines for "the care of the older or frail orthopaedic trauma patient" and the results of this adherence on clinical patient outcome measures.

Methods: This was a clinical audit. All ≥65-year-olds admitted to the orthopaedic department with a fragility fracture between 8 September 2022 and 8 March 2023 with a length of stay (LOS) of >72 hours were included. Patients were stratified into hip fracture (HF) and non-hip fracture (NHF) patients. A further similar cohort of NHF admissions between 8 March and 8 May 2023 was added to the data. The adherence of both cohorts to the national guidelines was recorded. Primary outcome measures of each cohort were recorded such as LOS and patient mortality.

Results: Data from 70 patients was collected. HF patients adhered to the guideline 79.4% of the time (31/39 patients) compared to NHF patients at only 19.3% of the time (6/31 patients) (p<0.001). Further, on average, HF patients were seen by an orthogeriatrician 15 times compared to just five times for NHF patients during their hospital stay (p<0.001). No significant difference in LOS or in mortality at 30 days post-admission was observed.

Conclusion: Medical orthogeriatric care is unequal despite similar LOS and mortality between both cohorts; thus, increasing orthogeriatrician input in NHF patients may lead to better patient outcomes for these patients.

## Introduction

Introduction

The elderly population within the United Kingdom has been increasing at a steady rate, with the number of people aged 65 or over increasing from 9.2 million to 11 million from 2011 to 2021, with an increase in the proportion of the population comprising this age group from 16.4% to 18.6% over the same time period [1]. This age group has been associated with worse outcomes after trauma with a 2.4-5.6 times greater risk of death, highlighting the need for adequate medical care in these patients [2].

Elderly hip fracture patients have a significantly increased risk of morbidity and mortality. Treatment of these patients consists of a well-defined surgical management plan; however, medical management of these patients is less clear-cut [3]. Due to this, the Italian Ministry of Health decided that elderly (>65) hip fracture patients should receive treatment within 48 hours [4].

Elderly hip fracture patients have proven difficult to manage. Although surgery within 24 hours has been shown to improve morbidity and mortality in young patients, there is evidence to show that elderly patients may suffer from early surgical intervention due to comorbidities making them less fit for surgery, highlighting the need for medical assessment in the early presentation period of these patients [5,6].

Outcomes of fragility fracture patients in the United Kingdom

Worldwide, approximately 200 million women have osteoporosis [7]. Fractures in the proximal femoral neck are the most sustained extremity fracture in elderly osteoporotic patients, followed by pelvic, femoral shaft, proximal humerus, distal femur, and distal radius fractures [8].

Fragility fractures have an increased morbidity and mortality, with some studies showing an increase in cardiovascular events in males following fragility fractures [9]. Outcomes in elderly trauma patients are believed to be worse than their younger counterparts due to their reduced physiological reserves to deal with ill health and aid recovery [10]. Pre-existing comorbidities such as osteoporosis, cardiovascular disease, respiratory disease, and endocrine disease may all complicate perioperative management in these patients [3,11]. This is reflected through prolonged hospital stays, diminished quality of life, and longer recovery times in these patients, as it has been reported that approximately 50% of elderly patients postoperatively require pharmacological management due to clinical deteriorations in the first few days following surgery [12].

Aims and objectives

The purpose of this study is to determine whether there is an adequate standard and/or disparity of care between two independent elderly trauma cohorts which comprise hip fracture (HF) and non-hip fracture (NHF) patients. Anecdotally, it was recognised that HF patients were being seen more by geriatricians than NHF patients; thus, it was deemed necessary to review the hospital's adherence to the British Orthopaedic Association's Standards for Trauma and Orthopaedics (BOAST) guidelines for "the care of the older or frail orthopaedic trauma patient" [13]. This is to ensure a standard of care which is equitable across patients with both cohorts. Our additional aim was to evaluate the patient outcomes across both cohorts and assess if these correlated with adherence to the BOAST guidelines. A correlation between the standard of care received and primary outcome measures in the form of patient mortality and length of stay (LOS) was also investigated to shed light on whether poor adherence to guidelines may be related to poorer outcomes.

## Materials and methods

Patients were identified from the online patient database at our hospital. The inclusion criteria identified retrospectively all ≥65-year-olds admitted to the orthopaedic department with a fragility fracture between 8 September 2022 and 8 March 2023 with a LOS of >72 hours (n=70). Patients were divided into two cohorts: HF and NHF patients. The HF group contained femoral neck, femoral shaft, and periprosthetic fractures (n=39) The NHF group contained fractures to any other part of the body (n=31). A further similar cohort of admissions between 8 March and 8 May 2023 was added to the data, filtering out HF admissions to provide a larger cohort of NHF patients that was similar in number to the HF cohort.

The standards assessed were whether patients were "seen by a geriatrician for a comprehensive geriatric assessment (CGA) commencing within 72 hours of injury" and "seen by a physiotherapist on postoperative day 1 with early identification of functional rehabilitation goals," as per the BOAST guidelines for "the care of the older or frail orthopaedic trauma patient" [13]. Primary outcome measures recorded from the hospital's online records system included LOS and patient mortality 30 days after admission. The total number of geriatrician reviews during the whole LOS for the patients from both cohorts was gathered, and the mean number of geriatrician reviews per patient was calculated for each cohort. Further confounding factors were investigated such as weekend presentation, and its effect on guideline adherence was investigated [13].

Percentage adherence to the guidelines in HF and NHF groups was calculated, respectively, and the difference in adherence was tested for significance by chi-squared testing. The percentage of patients overall presenting on a weekend versus a weekday was calculated, and chi-squared testing was used to determine if the day of presentation affected adherence. Chi-squared testing was further used to investigate the difference in adherence to BOAST physiotherapy guidelines and mortality rate between both cohorts. T-testing was used to assess the statistical significance between HF and NHF groups in the mean number of geriatrician reviews and mean LOS.

## Results

The total number of patients included in this study was 70, 39 in the HF cohort and 31 in the NHF cohort. The mean age was 81 (range 65-99) (SD=8.7). Table 1 summarises the population demographics of this study.

**Table 1:** Summary table of patient demographics

	Hip fracture cohort	Non-hip fracture cohort
Number of patients	39	31
Mean age	83	78
Mean length of stay (days)	28	22

**Figure 1:**
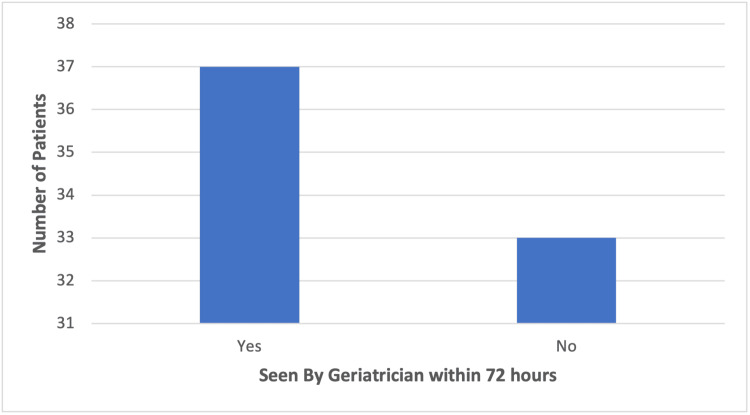
Bar chart demonstrating the number of patients fulfilling the BOAST criterion to be seen by a "geriatrician for a complex geriatric assessment commencing within 72 hours of injury" BOAST: British Orthopaedic Association's Standards for Trauma and Orthopaedics

Following analysis of the recorded data of the above cohorts, overall, only 37 (52.9%) of patients across both cohorts were seen by a geriatrician within 72 hours as per the BOAST guidelines. The adherence to the guidelines can be visualised below (Figure 1). Thirty-one out of 39 (79.5%) HF patients compared to six out of 31 (19.3%) NHF patients fulfilled this criterion. A chi-squared test showed this to be a statistically significant difference (p<0.001; Phi 0.598).

**Figure 2:**
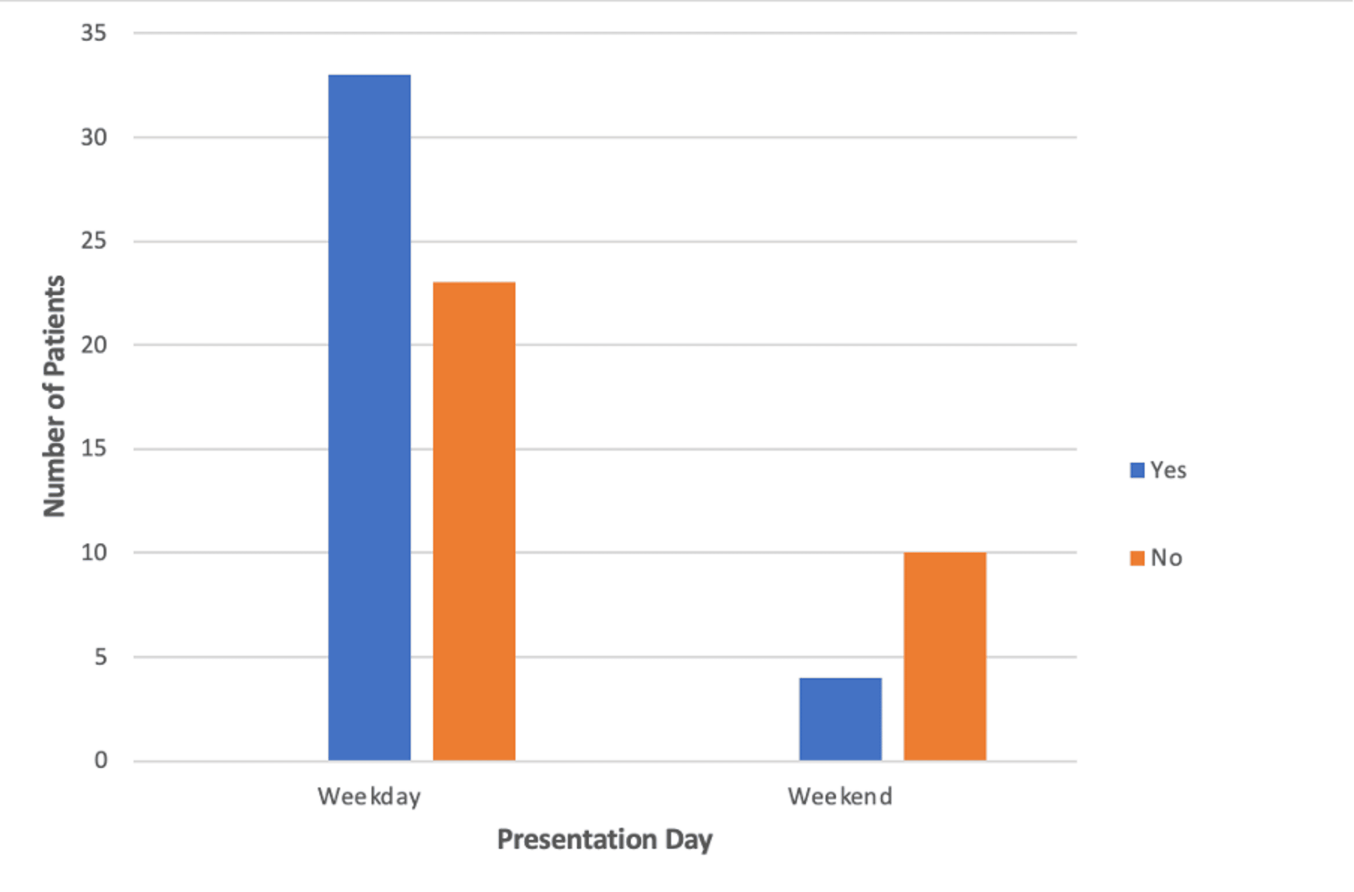
Bar chart demonstrating the number of patients seen by a geriatrician within 72 hours when presenting on a weekday or weekend

Thirty-three out of 56 (59%) patients who presented on a weekday were seen by a geriatrician within 72 hours, compared to four out of 14 (29%) patients presenting at the weekend (Figure 2). However, this demonstrated no statistically significant difference (p>0.01; Phi 0.243).

**Figure 3:**
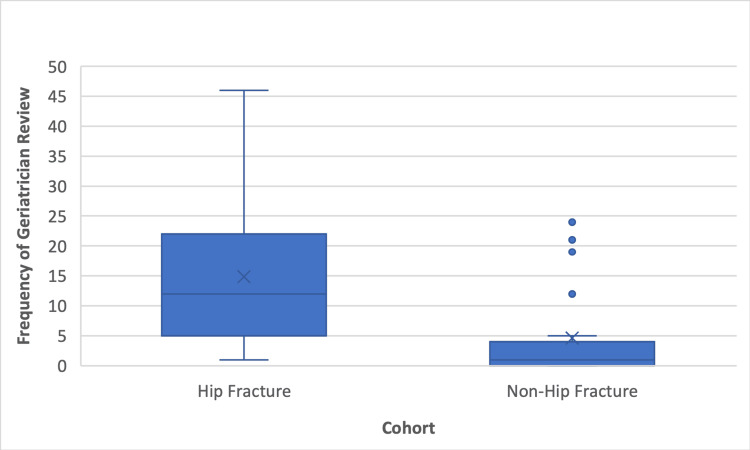
Box plot showing the number of times each cohort was reviewed by a geriatrician

The mean number of geriatrician reviews was 15 versus five for HF and NHF cohorts, respectively. A statistically significant difference was found on the t-test (p<0.001; 95% CI LL 5.5-UL 15.0). Figure 3 highlights the higher mean, median, and upper and lower values of the HF cohort compared to the NHF cohort and suggests a higher standard of care for HF patients.

**Figure 4:**
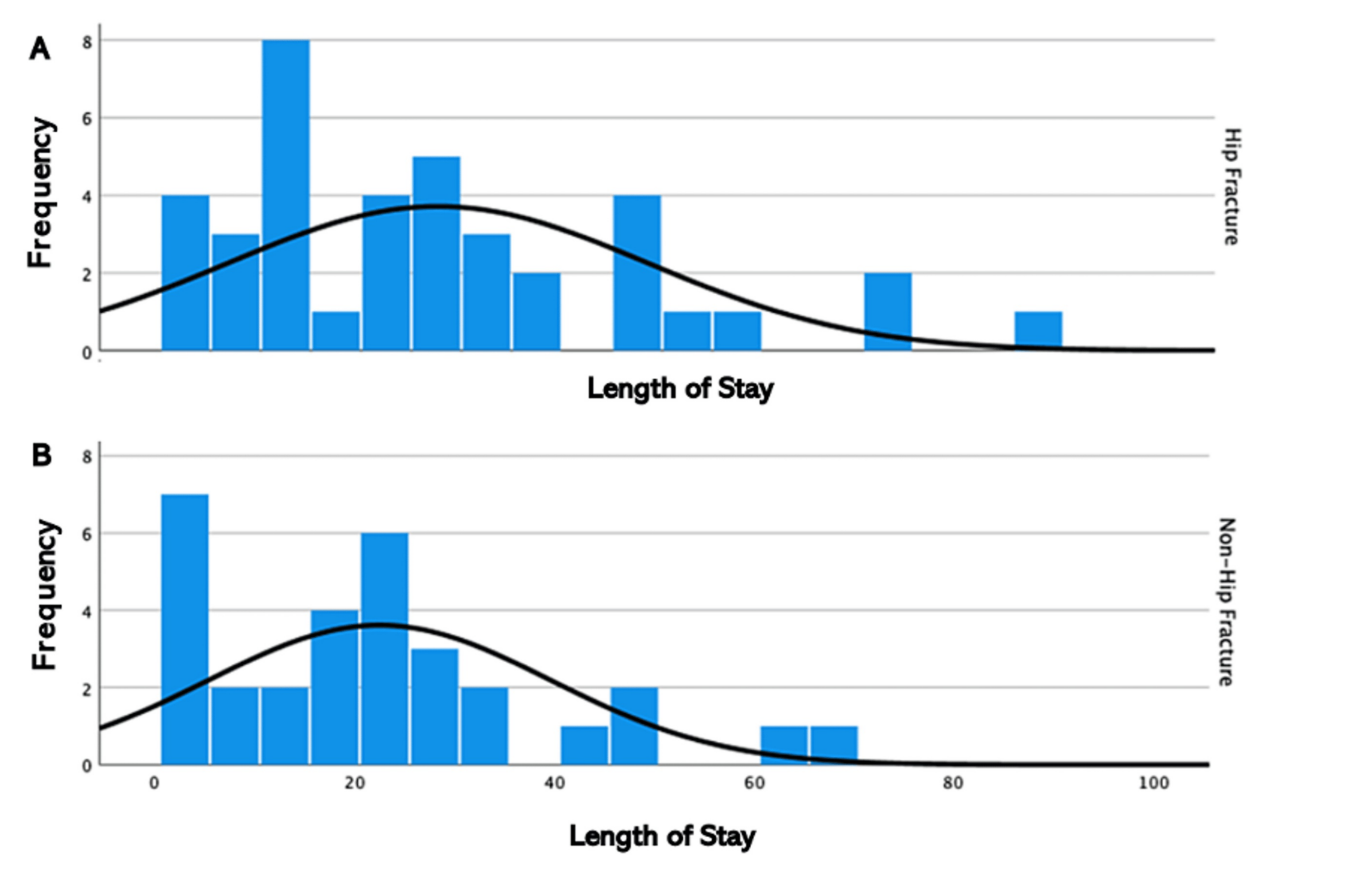
Histogram illustrating the length of stay for (A) hip fracture and (B) non-hip fracture patients

The mean LOS for the HF cohort was 28 days, compared to 22 days for the NHF cohort. This difference was not statistically significant (p>0.05), and the similarity of the LOS between both cohorts is illustrated below (Figure 4). Mortality rate between cohorts was also shown to have no statistically significant difference (p>0.05), as three of 39 (8%) HF and one of three (3%) NHF patients were deceased at 30 days from admission.

Of the patients who underwent operations, eight out of 16 (50%) NHF patients were seen within 24 hours by a physiotherapist, compared to 30 out of 36 (83%) HF patients fulfilling this criterion. This difference, however, was insignificant with p>0.05.

## Discussion

The elderly population has been increasing in recent years due to longer life expectancy. This has resulted in estimates that by 2050 there will be up to 6.3 million HF cases worldwide [11,14].

Elderly trauma is a significant cause of mortality in the United Kingdom, with up to a third of patients dying in the first year after injury and 8-10% within the first month [15]. However, similar figures are seen in elderly trauma across the spectrum, with a 25%, 35%, and 45% mortality rate seen in all elderly trauma patients in the age groups 70-79, 80-89, and 90+, respectively, in the first year post-discharge [16]. Despite modern treatment involving an extensive multidisciplinary team of physiotherapists, radiologists, nurses, and doctors, this is not believed to be enough due to the complex nature of managing elderly trauma patients [11]. An obvious solution to this issue is the provision of adequate collaborative care between geriatricians and orthopaedic surgeons throughout the LOS of an elderly trauma patient. This has been reported in numerous studies to improve mortality in patients who are managed with this approach compared to patients managed with no geriatrician, reducing LOS as well as being "cost-saving" as reported by NICE [11,17,18]. This is believed to be due to the enhanced medical management from geriatricians, which is tailored to the elderly patient's medical needs and minimises postoperative complications [19].

In this study, we found only 37 out of 70 (52.9%) elderly trauma patients met the BOAST guidelines of being seen by a geriatrician within 72 hours of presentation. This may have contributed to increased LOS and mortality, leading to poorer outcomes for patients at our hospital [13,18]. Interestingly, 31 out of 39 (79.5%) HF patients met this guideline compared to only six out of 31 (19%) NHF patients. This was demonstrated to be a statistically significant difference, and thus, there is a disparity in the frequency of medical review that NHF patients received compared to HF patients with similar clinical frailty scores (CFS). The guidelines state that "all patients admitted having sustained a fragility fracture and patients sustaining major trauma who have a CFS of 5 or more" should be "managed in a frailty pathway which includes comprehensive geriatric assessment commencing within 72 hours of injury" [13]. This difference was also reflected in the total number of geriatrician reviews, as HF patients were seen on average 15 times compared to five times for NHF patients throughout the full LOS. No significant difference was identified in the number of patients meeting this standard between those who presented on weekdays and those who presented on weekends. Due to the published correlation between improved outcomes and increased geriatrician input in elderly trauma patients, our results may indicate suboptimal treatment and subsequent poorer outcomes for patients who present to our hospital [17,18]. However, this was not reflected in our primary outcome measures, as there were no statistically significant differences in LOS or mortality between either cohort. This suggests that if the percentage of NHF patients receiving geriatrician review within 72 hours and the frequency of geriatrician review were to increase to the same level as HF patients, there may be an improvement in LOS and mortality rate for NHF patients.

Limitations to this study include the relatively low numbers in each cohort. Elderly patients were identified as being >65, but were not stratified into different age ranges to investigate the potential differences. Another limitation was that although the guidelines require patients to be included with a CFS >5, this was not recorded directly in the online hospital records system; thus, an estimation of the CFS of a patient was made from anaesthetic preoperative notes which described the patient's fitness for surgery. A regression analysis was not performed, which may have enhanced results. The data concerning the presentation of patients on the weekend versus weekdays may also have failed to show a clinically significant difference in the ability to meet the BOAST guidelines due to the small patient cohort of 14 patients.

## Conclusions

We identified a statistically significant difference in the percentage of patients receiving geriatrician review within 72 hours and the frequency of geriatrician review between HF and NHF patients in ≥65-year-olds with a similar CFS. NHF patients receive significantly less orthogeriatrician input at our hospital, highlighted by a significant disparity in mean visits throughout the patient's stay which may contribute to poorer patient mortality and morbidity. There was no difference in physiotherapy input between operative patients in each group. LOS and patient mortality were identified to be similar between HF and NHF patients, which may indicate an equal need in orthogeriatrician input, which may lead to further improvement in NHF patient outcomes. Meeting the BOAST guidelines in all elderly trauma patients will help ensure safe clinical practice and potentially improve patient outcomes for patients of all injury types.

## References

[1] (2024). Profile of the older population living in England and Wales in 2021 and changes since 2011. https://www.ons.gov.uk/peoplepopulationandcommunity/birthsdeathsandmarriages/ageing/articles/profileoftheolderpopulationlivinginenglandandwalesin2021andchangessince2011/2023-04-03.

[2] Grossman MD, Miller D, Scaff DW, Arcona S (2002). When is an elder old? Effect of preexisting conditions on mortality in geriatric trauma. J Trauma.

[3] Rolland Y, Abellan van Kan G, Bénétos A (2008). Frailty, osteoporosis and hip fracture: causes, consequences and therapeutic perspectives. J Nutr Health Aging.

[4] Moja L, Piatti A, Pecoraro V (2012). Timing matters in hip fracture surgery: patients operated within 48 hours have better outcomes. A meta-analysis and meta-regression of over 190,000 patients. PLoS One.

[5] Zuckerman JD, Skovron ML, Koval KJ, Aharonoff G, Frankel VH (1995). Postoperative complications and mortality associated with operative delay in older patients who have a fracture of the hip. J Bone Joint Surg Am.

[6] Bone L, Bucholz R (1986). The management of fractures in the patient with multiple trauma. J Bone Joint Surg Am.

[7] Cooper C, Campion G, Melton LJ 3rd (1992). Hip fractures in the elderly: a world-wide projection. Osteoporos Int.

[8] Court-Brown CM, Caesar B (2006). Epidemiology of adult fractures: a review. Injury.

[9] Paccou J, D'Angelo S, Rhodes A (2018). Prior fragility fracture and risk of incident ischaemic cardiovascular events: results from UK Biobank. Osteoporos Int.

[10] Perdue PW, Watts DD, Kaufmann CR, Trask AL (1998). Differences in mortality between elderly and younger adult trauma patients: geriatric status increases risk of delayed death. J Trauma.

[11] Aletto C, Aicale R, Pezzuti G, Bruno F, Maffulli N (2020). Impact of an orthogeriatrician on length of stay of elderly patient with hip fracture. Osteoporos Int.

[12] Folbert EC, Hegeman JH, Gierveld R, van Netten JJ, Velde DV, Ten Duis HJ, Slaets JP (2017). Complications during hospitalization and risk factors in elderly patients with hip fracture following integrated orthogeriatric treatment. Arch Orthop Trauma Surg.

[13] (2024). The care of the older or frail orthopaedic trauma patient. https://www.boa.ac.uk/static/a30f1f4c-210e-4ee2-98fd14a8a04093fe/boast-frail-and-older-care-final.pdf.

[14] Menzies IB, Mendelson DA, Kates SL, Friedman SM (2012). The impact of comorbidity on perioperative outcomes of hip fractures in a geriatric fracture model. Geriatr Orthop Surg Rehabil.

[15] Neuburger J, Currie C, Wakeman R (2015). The impact of a national clinician-led audit initiative on care and mortality after hip fracture in England: an external evaluation using time trends in non-audit data. Med Care.

[16] (2024). Major trauma in older people. https://www.gmccmt.org.uk/wp-content/uploads/2019/11/Major-Trauma-in-Older-People-2017-1.pdf.

[17] Van Heghe A, Mordant G, Dupont J, Dejaeger M, Laurent MR, Gielen E (2022). Effects of orthogeriatric care models on outcomes of hip fracture patients: a systematic review and meta-analysis. Calcif Tissue Int.

[18] Neuburger J, Currie C, Wakeman R (2017). Increased orthogeriatrician involvement in hip fracture care and its impact on mortality in England. Age Ageing.

[19] Greenstein AS, Gorczyca JT (2019). Orthopedic surgery and the geriatric patient. Clin Geriatr Med.

